# Predator-induced changes in the growth of eyes and false eyespots

**DOI:** 10.1038/srep02259

**Published:** 2013-07-25

**Authors:** Oona M. Lönnstedt, Mark I. McCormick, Douglas P. Chivers

**Affiliations:** 1 ARC Centre of Excellence for Coral Reef Studies, and School of Marine and Tropical Biology, James Cook University, Townsville, Qld4811, Australia; 2 Department of Biology, University of Saskatchewan, Saskatoon, SK S7N 5E2, Canada

## Abstract

The animal world is full of brilliant colours and striking patterns that serve to hide individuals or attract the attention of others. False eyespots are pervasive across a variety of animal taxa and are among nature's most conspicuous markings. Understanding the adaptive significance of eyespots has long fascinated evolutionary ecologists. Here we show for the first time that the size of eyespots is plastic and increases upon exposure to predators. Associated with the growth of eyespots there is a corresponding reduction in growth of eyes in juvenile Ambon damselfish, *Pomacentrus amboinensis*. These morphological changes likely direct attacks away from the head region. Exposure to predators also induced changes in prey behaviour and morphology. Such changes could prevent or deter attacks and increase burst speed, aiding in escape. Damselfish exposed to predators had drastically higher survival suffering only 10% mortality while controls suffered 60% mortality 72 h after release.

Colour patterns are often adaptations to ecological pressures, and the sheer diversity of patterns represents an important form of morphological evolution in animals^1^. Many terrestrial insects, especially lepidopterans, as well as marine and freshwater fishes are often characterized by one or several conspicuous eyespots present on less essential regions of the body^2^,^3^. False eyespots are large, dark circles surrounded by a lightly coloured ring thought to represent an iris around a pupil, mimicking the appearance of a vertebrate eye. The adaptive significance of false eyespots in prey has long been debated among ecologists. Decades of research have led to four hypotheses regarding their function, and their presence has been attributed to - deterring predators (intimidation hypothesis^4^, as a diversion technique drawing the attacks of predators to non-vital regions of the body (deflective hypothesis^5^), a form of status signalling (i.e., status signalling hypothesis^6^) or simply as an evolutionary remnant no longer utilized^7^.

Due to the widespread occurrence of eyespots in a variety of unrelated taxa, these ‘false eyes' are believed to have evolved in response to selective pressures^3^. Powell^8^ found that the conspicuous black tail tip (thought to mimic an eye) on long-tailed white weasels (*Mustela frenata*) reduces predation by avian predators. Hawks attacking white weasel models in snowy environments were more likely to become confused and attack the conspicuous tail tip, often missing their target. Similarly, Blest^4^ and Smith^9^ found that predators were more likely to direct their attacks toward conspicuous eyespots that had been painted on insect prey. It appears as if colour patterns that mimic eyes may be an effective deflection mark for many different prey species, although the adaptive significance of this has yet to be tested. Predators have been found to trigger striking changes in growth and morphology in a variety of prey (e.g. body depth^10^,^11^), but whether presence of predators influence the development of prey eyespots has never been tested.

In addition to triggering morphological defences cues from predators and/or injured conspecifics also affect prey behaviour. The presence of consumers induce ‘anti-predator behaviours' in prey, such as reduced foraging, lowered activity and increased refuge use^12^. These behavioural defences will ultimately influence the prey's success by altering the balance between defensive behaviours and other activities that promote fitness. The relative importance of predator cues in influencing behaviour and survival of prey has received attention in a number of studies^12^,^13^, but few studies have looked at how different predation cues simultaneously affect prey development, colour patterns and behaviour over an extended period of time (but see^14^). Reducing predation through behavioural and physiological means could potentially increase short-term survival but may also result in lowered overall fitness and reduced survival in the long-term.

Juvenile damselfish have lightly coloured bodies and a conspicuous eyespots on the rear dorsal fin, which fades away as individuals approach maturation. Damselfish are an abundant component of the Great Barrier Reef fish community, with high vulnerability to predation during recruitment^15^, and represent a useful organism with which to explore how growth and colour patterns are affected by the continuous exposure to predators, and how these changes may confer a survival advantage to individuals in their natural environment. The current study therefore explored how threat cues from a common predator, *Pseudochromis fuscus*, indirectly affected development and performance of a juvenile damselfish, *Pomacentrus amboinensis*. Specifically, we tested how the continuous exposure of individual prey to a predator affected prey morphology (body depth, BD; standard length, SL), eyespot size (total diameter), total visible size of the eye and behaviour over a 6-week period, after which survival patterns in the field were monitored. Usually, *P. amboinensis* will lose their eyespots as they age^7^, but we hypothesized that if eyespots evolved as a defence against consumers then the continuous exposure of prey to predators would result in the continued growth of the eyespot.

## Results

### Differences in morphology among treatments

**Figure 1:**
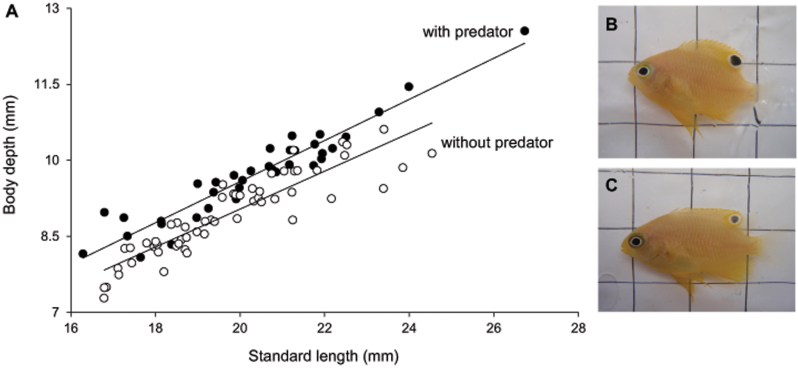
Comparison of depth to length ratio. The relationship between standard length (SL) and body depth (BD) *of P. amboinensis* when in the presence and absence of predators (A). Fish had significantly deeper bodies when exposed to predator cues (B) compared to the shallow bodied controls (C).

**Figure 2:**
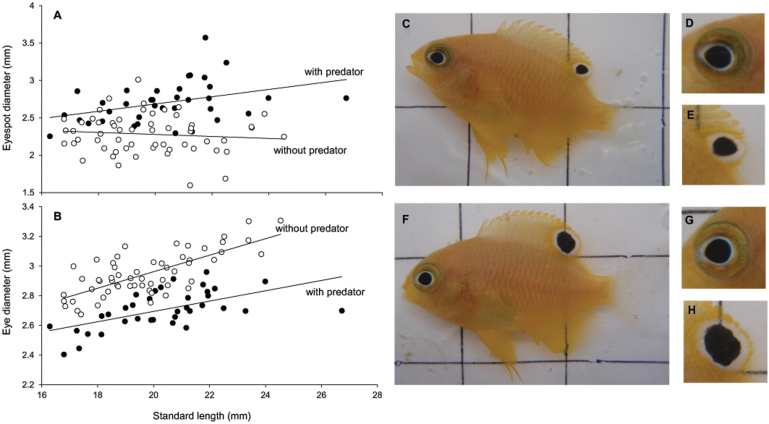
Relationships between eyespot size and eyeball size and body length. The relationship between standard length and eyespot diameter (A) and standard length and eye diameter (B) in presence and absence of predators. All prey fish exposed to predator cues over a 6 week period had significantly larger eyespots (F,H) and smaller eyes (F,G) than fish from the control treatments (C–E).

At week 0 there was no difference in morphological measurements (ANCOVA with standard length as covariate; body depth F_2, 89_ = 0.93, p = 0.09; size of ocellus F_2, 89_ = 0.47, p = 0.63; eye diameter F_2, 89_ = 0.65, p = 0.52;) among fish from the three different treatments. After 6-weeks, prey that had been exposed to predator cues had significantly deeper bodies for any given length than fish from the two control treatments (F_2, 89_ = 33.14, p<0.001; Fig. 1a–c) which, in turn, did not differ from one another (F_1, 54_ = 2.24, p = 0.14). Eyespot size (total diameter) was significantly different among treatments after a 6-week period, with prey exposed to predator cues having significantly larger eyespots for any given body length compared to the control treatments (SL: F_2, 89_ = 25.67, p<0.001; Fig. 2a), which did not differ from one another (F_1, 54_ = 0.19, p = 0.077). The visible part of the eye was also significantly different depending on treatment, with prey from predator treatments having significantly smaller eyes than individuals from the control treatments (F_2,89_ = 70.67, p <0.001; Fig. 2b). There was no difference in eye size between the 2 control treatments (F_1,54_ = 0.13, p = 0.72).

### Differences in behaviour among treatments

**Figure 3:**
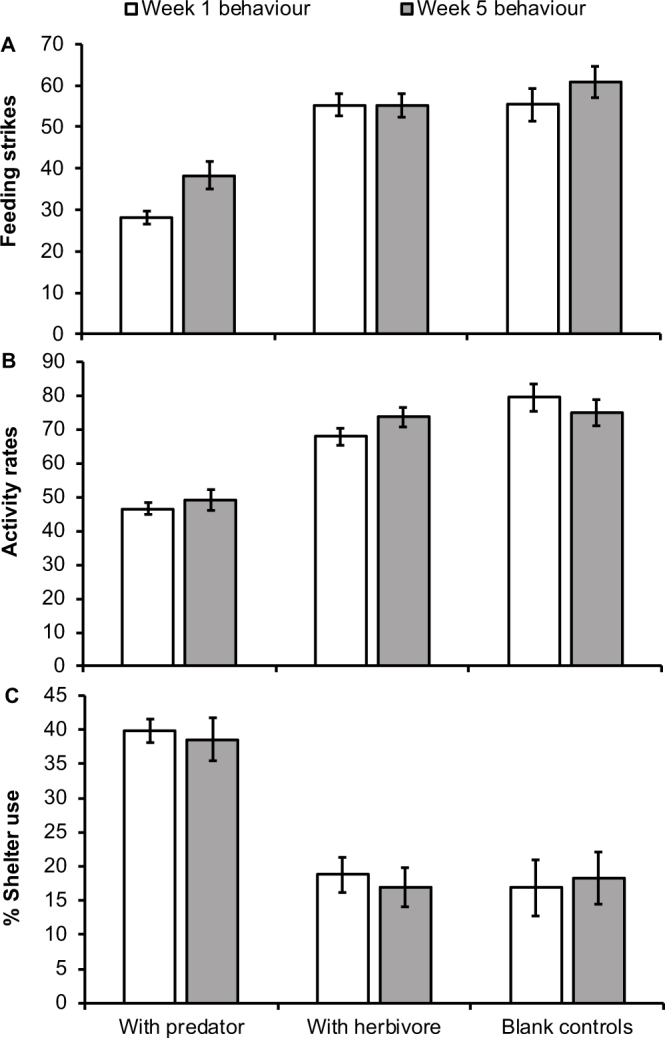
Predator presence influences prey behaviour. Fish exposed to predators foraged significantly less (A), displayed lower activity rates (B) and a significant increase in shelter use (C) compared to fish from the two control treatments after 1 week in the tanks. This pattern remained similar after 5 weeks. Bars are the standard errors around the mean from behavioural variables.

The multivariate analysis of variance revealed significant overall differences in behaviour depending on treatment after 1 week (MANOVA, F_3, 88_ = 12.41, p<0.0001). Univariate ANOVAs demonstrated that fish from the predator treatment foraged significantly less (F_2,90_ = 38.36, p<0.000; Fig. 3a), were less active (F_2,90_ = 19.58, p<0.0001; Fig. 3b) and spent more time in shelter (F_2,90_ = 29.10, p<0.000; Fig. 3c) compared to individuals in the herbivore treatment and the seawater control after 1 week. After 5-weeks there was still a significant differences in overall behaviour of fish (MANOVA, *F*_3,88_ = 5.67, p<0.001). Bite rate (F_2,90_ = 12.6, p<0.0001; Fig. 3a) and activity (F_2,90_ = 12.09, p<0.0001; Fig. 3b) were significantly lower and time in shelter was significantly higher (F_2,90_ = 16.49, p<0.0001; Fig. 3c) in fish exposed to predators than in fish exposed to herbivores or isolated.

### Differences in survival among treatments

**Figure 4:**
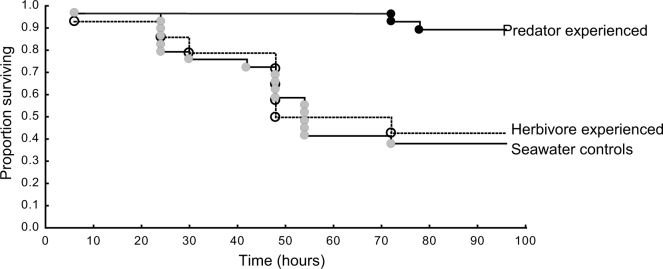
Survival patterns of fish from the three treatments. Survival curves (Kaplan Meier plot) of *P. amboinensis* in the field after laboratory exposure to predator cues, herbivore cues or no cues for a 6-week period. Fish were placed on small patch reefs along the edge of a reef and their survivorship was monitored 2 times a day for 4 days. Fish from predator treatment had the highest and similar survival.

Survival of prey when released in the field was affected by treatment (χ^2^_2,0.05_ = 19.88, p<0.001; Fig. 4). Patterns of survival were established within the first 48 h after release. Treatments split into two groups, with one group containing fish that had experienced the herbivores for 6-weeks (40% were consumed within 48 hours) and fish from the seawater treatments (50% were consumed within 48 hours), all with similar and low survival. The second group contained fish that had experienced predators for 6 weeks, with high survival rates following release (no fish had been consumed after 72 hours and 89% of fish were still alive after 96 hours).

## Discussion

Here we show that the presence of a predator induced significant changes in morphology, colour patterns and behaviour in a juvenile damselfish. Prey exposed to predators for 6-weeks grew deeper bodies, developed larger eyespots and exhibited stunted eye growth compared to prey exposed to herbivores or those that were isolated from other fish. The increase in body depth has been found in previous studies and is considered a common prey response to gape limited predators in a multitude of freshwater taxa^10^,^11^,^16^,^17^. What is intriguing is the finding that juvenile prey grow larger eyespots and display smaller eyes when continuously exposed to predators. The large eyespot in the caudal area of prey taken together with the smaller eye in the head region give an impression of the true eye being present in the posterior end of the body, potentially confusing predators about the orientation of prey. Predators anticipate the direction prey will move as an attack is initiated, and a false eyespot may aid prey by causing the predator to misjudge the direction of the prey's escape^8^. Also, a prey attacked at the invulnerable caudal area can escape and survive^8^,^18^, however an attack on the head would damage vital parts allowing almost no chance of survival. McPhail^18^ demonstrated that caudal spots in a characid fish (*Hyphessobrycon panamensis*) deflect the aim of the characinoid predator *Ctenolucius beani*, as the majority of predators focused their attacks on the caudal area of prey fish that had an artificial caudal spot drawn on compared to fish with no spot. Clearly, prey with artificial eye spots escape predators more frequently than prey with no eyespots^4^,^9^,^18^.

Findings from the current study suggest that false eyespots may be a direct short-term adaptation to the presence of predators, functioning to misdirect predator strikes and/ or protect the head region from fatal attacks. If the increased growth of a larger false eyespot is associated with a cost such as the development of smaller eyes (and possibly poorer vision) it would only be advantageous to develop this type of anti-predator mechanism in certain circumstances, such as in predator rich environments. Flexibility and degeneration in eye growth has been found in other teleost fishes^19^, most notably in the Mexican cavefish *Astyanux mexicanus*^20^,^21^. This species has 2 morphological variations, a surface-dweller with pigmented eyes, and several different eyeless and depigmented cave-dwellers^20^. It is evident that eye development is plastic and can evolve to suit certain environmental conditions, indeed in many young animals it is the visual stimuli received that influences eye growth patterns^19^. Ours is the first study to document predator-induced changes in the size of eyes and eye-spots in prey animals, however, others have documented that predation can result in selection for reduced eye pigments. For example, when comparing eye diameters in populations of the cladoceran, *Bosmina longirostris*, Zaret and Kerfoot^22^ found that prey living in areas associated with predators had significantly smaller eye-pigmentation diameter than *B. longirostris* from non-predation areas. They argue that fish predators select prey based on eye pigmentation area, and prey found in predator rich areas have evolved smaller eyes to minimize the probability of being caught.

Prey exposed to predators displayed more conservative behaviours, which included lower foraging rates, more time spent in shelter and reduced activity. Cautious behaviours remained largely intact even after 5 weeks in the predator treatment. The unchanged behaviours highlight the ecological relevance and importance of the predator stimulus. Reduced activity levels increases prey survival by making the prey less conspicuous to the predator^23^. Reduced activity also saves energy, allowing individuals to allocate more into growth and/or development of predator-induced morphological defences^24^. The mere presence of predators is enough to suppress activity of prey, and it has recently been suggested that this lowered activity is responsible for the increased growth of fish as the energy conserved in the presence of predators is allocated to growth^25^.

Predator experience and subsequent morphological changes confer a survival advantage to prey in their natural environment, as predator experienced prey with larger eyes spots and deeper bodies had drastically higher survival when stocked in the wild with control treatments suffering a 5 fold increase in mortality after 72 h on the reef. Results emphasize the importance of experience with predators to prey survival early on in life. The behavioural anti-predator response allows reduced detection by predators and the morphological defence and changed colour patterns may allow an improved ability to escape an attack. Deep bodies not only protect prey fish from gape-limited predators by deterring attacks^10^ but have also been found to improve speed, acceleration and manoeuvrability in both fish and amphibians^26^,^27^. This is the first study to provide direct empirical evidence that eyespot size is increased upon exposure to predators. Predators also stunt eye growth, as there is reduction in the relative eye diameter over time. These morphological changes likely direct attacks away from the head region, protecting the more vulnerable regions of the body. Our results illustrate how phenotypically plastic development in prey morphology and coloration as well as conservative behaviours can result in dramatic increases in survival.

## Methods

### Study organisms and collections

The study was conducted from October through to December 2010 In the laboratory facilities and reefs around Lizard Island Research Station (14°38′S, 145°28′E) on the northern Great Barrier Reef, Australia. Settlement stage damselfish (family Pomacentridae) were collected from light traps that had been deployed overnight about 50 m from the reef edge. The study species, *Pomacentrus amboinensis*, is an abundant and very common damselfish species that settles on the reefs during the summer months after a pelagic larval phase of 15–23 days^28^. Light traps catch the fish at the end of their larval phase, as they are entering the reefs at night to settle, therefore ensuring fish are naïve to reef-based, bottom-dwelling predators. Within 6 hours of settlement *P. amboinensis* will metamorphose and lose the transparent colour typical of the pelagic larval stage and gain the bright yellow body coloration and conspicuous black dorsal eye spot representing the juvenile stage of this species^29^. The predator used as the stimulus was the dusky dottyback, *Pseudochromis fuscus*, which is one of the most abundant meso-predators on the shallow reefs throughout the Indo-Pacific^30^. This particular species is responsible for consuming a large amount of the newly settled and juvenile damselfish during the summer recruitment season^31^, and is found in areas where *P. amboinensis* settle. A herbivorous goby, *Amblygobius phalanea*, was used as an experimental control to test for the effect of exposing *P. amboinensis* to visual and chemical cues of any heterospecific fish^32^. This fish has a similar body shape and size to the predatory dottyback and is often found in areas of the reef were recruits settle. Both species were caught on the reefs surrounding Lizard using a dilute clove oil anaesthetic and a handnet. Research was conducted under James Cook University ethics approval A1593 and A1720.

### Laboratory study and experimental design

Individual *P. amboinensis* were exposed to a combination of olfactory and visual cues of a predator (*P. fuscus*), a non-predator (*A. phalanea*) or a blank control (receiving no cue sources). The growth, development and behaviour of *P. amboinensis* were assessed over a 6 week period. Naïve prey fish that had been collected with light traps (were brought back to the laboratory and placed in 60 L flow-through tanks (density: 50fish/tank) over a period of 10 days and fed *Artemia* nauplii *ad libitum* 3 times per day (ensuring all fish used in the experiment had an analogous baseline body condition at the start of the experiment). All *P. amboinensis* individuals were then conditioned to recognize the sight and olfactory cues of *P. fuscus* by placing the predator inside a transparent plastic bag in their tank for 30 minutes, while simultaneously injecting previously collected odour cues of the predator and skin extract cues of *P. amboinensis*. This is a training procedure found to increase the probability of survival in the ambon damselfish^33^, and is necessary to make sure that prey can recognise the cues of the predator species. It also ensured that all fish had the same baseline predator experience before the commencement of the study. Individual prey then had their morphology and shape photographically recorded against a scale before being transferred into a series of specially-designed 18 L PVC predator–prey tanks (64.2 × 11.5 × 18 cm). The tanks had a 7.5 L main section (containing either a predator or a herbivore) and 6 individually isolated prey compartments (1.5 L: 10.7×13×18 cm). The main compartment was separated from each of the 6 prey compartments by transparent Perspex that contained a series of small holes. The fish in the six prey compartments were visually isolated from each other using grey PVC partitions. Water flowed from the main predator/herbivore compartment to each of the prey compartments and then out the side of each of the prey compartments. This arrangement ensured that the prey fish in each of the six compartments were also chemically isolated from one another (see supplementary material, Fig. S1). The bottom of both the predator/herbivore compartment and the prey compartment was covered by a 1.5 cm layer of sand and the predator/ herbivore section had one plastic tube (12×5 cm) placed in the centre to provide shelter. A small coral skeleton (*Pocillopora sp.* ~4×5×5 cm) was placed at the back of each prey compartment to provide a refuge. The tanks were situated outside to ensure that animals received all natural temporal cues and the water was supplied by a flow through system from the ocean so organisms were given all the same environmental cues as that of fish residing in the wild. This design ensured that the individual prey in each compartment received all the olfactory diet cues as well as visual cues from the main section, ensuring all prey could both smell and see either the predator or herbivore (n = 36 fish/treatment), but that the prey could not see or smell each other. The chemical and visual isolation allowed us to consider the fish in each compartment as independent samples. Prey were fed twice daily with a standardized amount of boosted (DHA Selco) *Artemia* sp*.* nauplii (5 ml with ~550 *Artemia*/ ml) while predators were fed two damselfish individuals morning and night, which is an accurate representations of what *P. fuscus* consume in their natural environment^31^ ensuring that the cue stimulus provided to *P. amboinensis* was realistic. Gobies were given a combination of dry fish food pellets (INVE Aquaculture Nutrition NRD pellets; containing no fish products) and small crustaceans. Predators and herbivores were replaced every weeks, ensuring that significant effects could not be attributed to individual predators/herbivores. In addition to this there was an experimental control were individual prey were placed in separate 1.5 L compartments (10.7×13×18 cm) that received no cue sources (n = 21). After 6 weeks individual *P. amboinensis* were removed from their compartments and photographed against a scale (10×10 mm) for morphological measurements. Shape and size of fish were analysed from digital photographs using the software *Optimas 6.5*. Five variables were measured: standard length, body depth, total area of ocellus, diameter of ocellus (black and white), and entire diameter of the visible eye.

### Monitoring prey behaviour

One week after the commencement of the experiment, a mirror (80 × 40 cm) was suspended over each tank at 45° so that focal fish could be observed undisturbed from above. A wire grid (2×2 cm) was also placed on the top of each chamber so that movement and location of individuals could be accurately quantified as the number of times fish crossed a line on the grid. Water flow was stopped and individual *P. amboinensis* were fed *Artemia sp.* nauplii. One minute later the fish had their behaviour assessed for a 2 min period. The mirror and grid were then removed. This procedure was repeated after 5 weeks for all treatments. The behaviour of individual fish in each of the 7 experimental treatments was quantified by recording: total number of feeding strikes (successful or otherwise), activity (quantified as the number of times a fish crossed a line on the grid that had been suspended over the tank), and % time spent within shelter (defined as being inside the branches of the coral shelter).

### Field survival

After being photographed prey fish from each treatment were transferred onto individual patch reefs in the field. Patch reefs (25×15×20 cm) were placed 2 meters away from the main reef and 3 metres apart and were made up of healthy *Pocillopora damicornis* colonies (a hard bushy coral), which is the preferred settlement site for *P. amboinensis*. Individual fish were transferred onto separate patch reefs and left to acclimate with a cage on top for 1 h, before having the cage removed (sample size ranged from 14–27 per treatment). Following the acclimation time, individual fish had their survival monitored twice a day (morning and afternoon) for 4 days after release by SCUBA divers^12^. Fish were assumed to be caught by a predator when missing from the patch reef. Cage controls that allowed fish to swim away found that there was no movement from patches, suggesting that when a fish was missing it was due to predation rather than migration.

## Author Contributions

O.M.L. and M.I.M. contributed to the design of experiments. O.M.L. collected, reared and monitored the animals and carried out the field work. O.M.L. and M.I.M. contributed to the analyses and interpretation of data. O.M.L. drafted the paper and M.I.M. and D.P.C. substantially contributed to paper revisions.

## Supplementary Material

Supplementary Information Supplementary information
