# Supplementary material for: Predator-induced changes in the growth of eyes and false eyespots
Source: Sci Rep. 2013 Jul 25;3:2259. doi: 10.1038/srep02259 (PMC3722912; doi:10.1038/srep02259)
Supplement: Supplementary Information [file srep02259-s1.pdf]

Supplementary Information

Predator-induced changes in the growth of eyes and false eyespots

Oona M. Lönnstedt[1]*, Mark I. McCormick[1], Douglas P. Chivers[2]

[1] *ARC Centre of Excellence for Coral Reef Studies, and School of Marine and Tropical Biology, James Cook University, Townsville, Qld4811, Australia*

[2] *Department of Biology, University of Saskatchewan, Saskatoon, SK S7N 5E2, Canada*

*To whom correspondence should be addressed. Email: oona.lonnstedt@my.jcu.edu.au

This file includes

Supplementary Figure S. Experimental tank Design

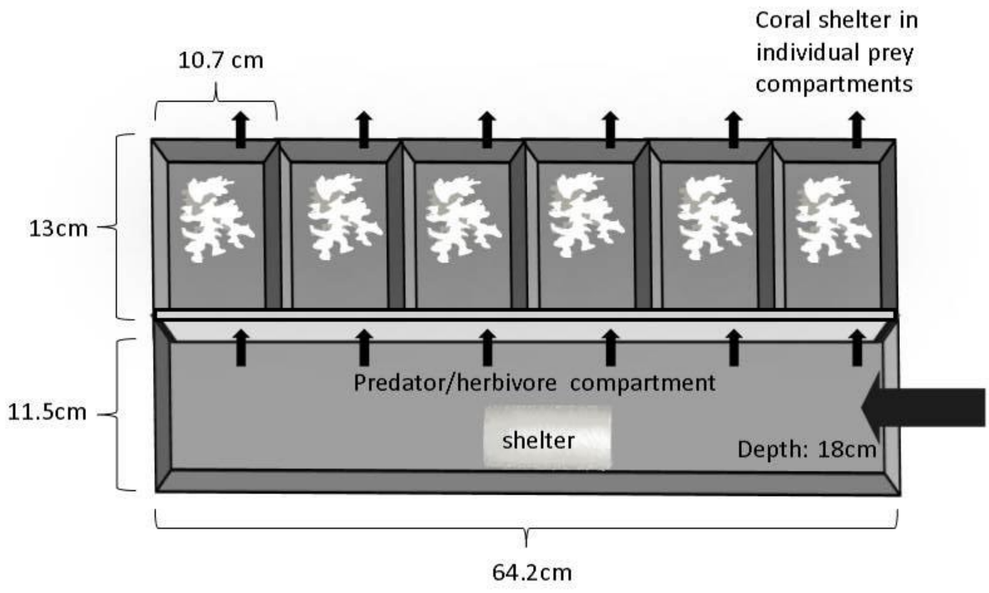

Supplementary Figure S1. Design of the combined senses tank where prey received both olfactory and visual cues. Prey fish were placed in individual compartments containing a coral skeleton (shelter). These individual prey compartments were adjacent to one longer compartment containing a small shelter and either a predator or a herbivore (or no fish). The partitioning dividing the small compartments from the large one is transparent Perspex with small holes to allow the water through. Arrows indicate direction of water flow; the main section received the flowing water while small holes in the side of the compartments ensured the water flowed into the individual prey compartments and out through holes in each individual room (ensuring that each compartment received olfactory diet cues from the main section, and that the prey compartments were chemically isolated from each other). Individual prey compartments were separated from one another by opaque grey PVC barriers that were sealed in place (ensuring prey where visually isolated from one another). The chemical and visual isolation of each compartment allowed us to consider each prey compartment as an independent replicate (n=36 fish per treatment).
